# *Pseudomonas aeruginosa *contamination of mouth swabs during production causing a major outbreak

**DOI:** 10.1186/1476-0711-6-3

**Published:** 2007-03-13

**Authors:** Bjørn G Iversen, Hanne-Merete Eriksen, Gjermund Bø, Kristian Hagestad, Trond Jacobsen, Eva Engeset, Jørgen Lassen, Preben Aavitsland

**Affiliations:** 1 Division of Infectious Disease Control, Norwegian Institute of Public Health, Oslo, Norway; 2 The Norwegian Food Safety Authority, district office of Vest-Agder, Kristiansand, Norway; 3 The Norwegian Board of Health in the County of Vest-Agder, Kristiansand, Norway; 4 St. Olavs Hospital, Trondheim, Norway

## Abstract

**Background:**

In 2002 we investigated an outbreak comprising 231 patients in Norway, caused by *Pseudomonas aeruginosa *and linked to the use of contaminated mouth swabs called Dent-O-Sept. Here we describe the extent of contamination of the swabs, and identify critical points in the production process that made the contamination possible, in order to prevent future outbreaks.

**Methods:**

Environmental investigation with microbiological examination of production, ingredients and product, molecular typing of bacteria and a system audit of production.

**Results:**

Of the 1565 swabs examined from 149 different production batches the outbreak strain of *P. aeruginosa *was detected in 76 swabs from 12 batches produced in 2001 and 2002. In total more than 250 swabs were contaminated with one or more microbial species. *P. aeruginosa *was detected from different spots along the production line. The audit revealed serious breeches of production regulations. Health care institutions reported non-proper use of the swabs and weaknesses in their purchasing systems.

**Conclusion:**

Biofilm formation in the wet part of the production is the most plausible explanation for the continuous contamination of the swabs with *P. aeruginosa *over a period of at least 30 weeks. When not abiding to production regulations fatal consequences for the users may ensue. For the most vulnerable patient groups only documented quality-controlled, high-level disinfected products and items should be used in the oropharynx.

## Background

*Pseudomonas aeruginosa *is a gram-negative, obligate aerobe rod-shaped bacterium with minimal nutritional requirements. It is often found in moist environment and can cause infections in immunocompromised or otherwise susceptible hosts [1,2]. Numerous outbreaks have been associated with faulty or unclean medical equipment or products [3-9], contaminations from personnel or environmental reservoirs [10-16]. Cross-colonization and cross-contamination within hospitals has been documented [13,17,18].

We have reported a major, nationwide outbreak of *Pseudomonas aeruginosa *infection in 24 Norwegian hospitals [19]. The outbreak comprised of 231 patients with a genotypically identical strain of *P. aeruginosa *from the period November 2000 to December 2002, of which 39 were blood culture positive. Seventy-one infected patients, all of whom had severe underlying diseases, died while hospitalized. The outbreak strain was susceptible to all anti-pseudomonas antibiotics (ceftazidime, ciprofloxacin, imipenem-cilastadine and tobramycin). However, some of the isolates cultured late in the outbreak had developed intermediate susceptibility or full resistance to ceftazidime or aztreonam (MIC 96 and 24 mg/L, respectively).

**Figure 1:**
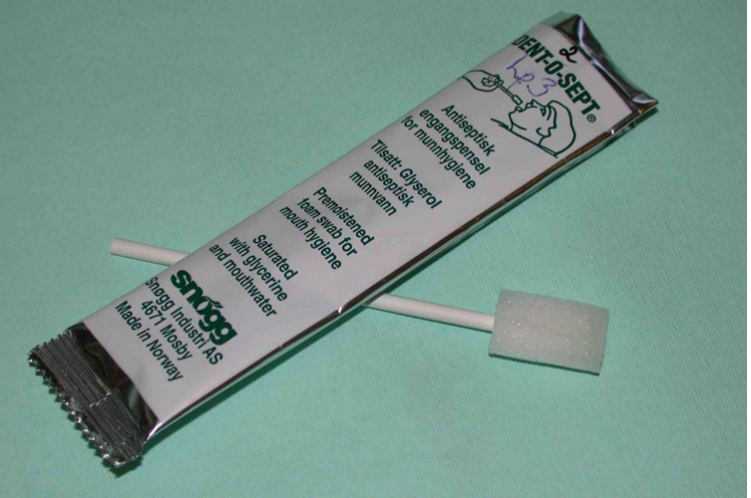
The Dent-O-Sept mouth swab. The English text on the wrap reads: "Premoistened foam swab for mouth hygiene. Saturated with glycerine and mouthwater".

The outbreak strain of *P. aeruginosa *was traced to a mouth swab called Dent-O-Sept. This is a clean, non-sterile, moist sponge-on-a-stick produced in Norway, which according to the Norwegian text on the wrap is an antiseptic single-use swab for mouth hygiene (Figure 1). (The English text on the wrap does not contain the word *antiseptic*.) This swab was the dominant product of its kind on the Norwegian market, being widely used in hospitals, long-term care facilities and in home care. Approximately one million swabs were sold in Norway per year. Small quantities were also exported to Denmark and Sweden. As soon as the connection between the swab and the outbreak was identified the company ceased production at its single facility and recalled the product.

The objective of this study is to examine how *Pseudomonas aeruginosa *contaminated the product, assess the extent of the contamination and identify critical points in the production process that made the contamination possible.

## Methods

### Setting

Norway has a population of 4.5 million people and approximately 65 general hospitals and around 1000 health care institutions for the elderly. There are 22 medical microbiological laboratories in the country providing general bacteriological culturing services. Through the European Economic Area Agreement Norway abides by much of the legislation within the European Union, including European Council Directive 93/42/EEC concerning medical devices [20].

### Investigation of contaminated product

The Norwegian Institute of Public Health (NIPH) coordinated the national outbreak investigation. Immediately after the recall of the product, we asked all hospitals to report to NIPH which batches of the Dent-O-Sept swab they had in store. The rest of the health care services were asked in a newsletter from NIPH to do the same. A batch number printed on the wrap indicated the week and year of production. Up to 10 swabs of each available batch of the product were examined at the microbiological laboratory which the health care institution normally used. We asked the laboratories to identify and deep freeze monocultures of all findings of gram-negative rods, *Staphylococcus aureus*, streptococci and enterococci. Other microbes like those often included in gram-positive mixed flora (micrococci and coagulase negative staphylococci) and *Bacillus *spp. were to be noted and reported.

### System audit and additional investigations

The Directorate for Health and Social Affairs organized a system audit of the manufacturer on 12 – 15 April 2002 by studying documents, interviewing selected personnel and inspecting the premises, including microbiological sampling of tap water, swabbing of different places along the production lane and culturing of stored and packed samples of the product. These samples were cultured at the municipal Food Control Authority. Isolates of *P. aeruginosa *were sent for genotyping as described below.

On request from the producer Snøgg Industri AS, the laboratory at the municipal Food Control Authority performed environmental sampling in addition to what had been performed during the system audit described above. The production site had been left untouched after the production had ceased on 9 April 2002. In May, quantitative analysis of *P. aeruginosa *was performed on the moisturizing liquid of 16 wrapped swabs taken from four boxes with swabs produced on the same day and from 15 swabs from three boxes with swabs produced at different times during two consecutive days.

**Figure 2:**
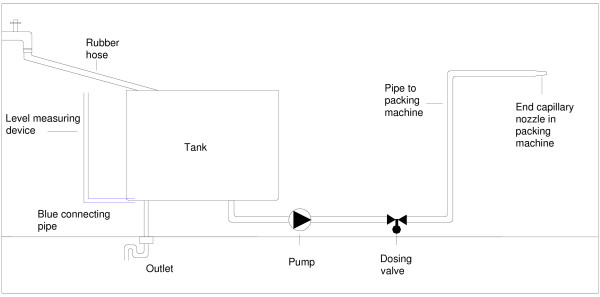
Schematic figure showing the wet part of the production of the Dent-O-Sept swab.

From the bottom of the steel tank a blue pipe connects to a level measuring device (Figure 2). In May 2002 samples were taken directly from remaining water in the blue connecting pipe and from water flushed through the level measuring device.

Between 28 May and 5 June 2002 water samples were taken from water taps located several places on the production site and from a 1 m chipped, rubber hose leading from a faucet with municipal water to the steel tank The hose had not been replaced in an estimated seven years. Quantitative analysis of *P. aeruginosa *was performed [21].

### Moisturizing liquid

We performed microbiological analysis [22,23] of each of the ingredients for the moisturizing liquid used in the production of the Dent-O-Sept swab (except tap water). The total viable aerobic count and specific detection of *P. aeruginosa *were tested in each of the liquids. Then the moisturizing liquid undiluted and in 1:10 dilution were tested for their effect on the outbreak strain and a reference strain of *P. aeruginosa *(ATCC 9027 -MicroBioLogics) [24].

### Microbiological analyses

Culturing of samples of Dent-O-Sept swabs was performed at local laboratories according to our instruction: "Brush the swab against both a lactose and blood agar dish in a rotating manner so all sides of the foam tip touches the agar. It is not necessary to place the swab in a growth broth". The isolates were identified by standard procedures in use by the laboratories.

Culturing of samples from the system audit and the additional investigation of the production site were performed at the laboratory of the municipal Food Control Authority. The qualitative analysis of the samples was performed by direct seeding (except for dry Dent-O-Sept swabs) and seeding after enrichment overnight in a heart infusion broth on Kings Agar B and on blood agar. The quantitative analysis was performed by direct seeding of 0.1 mL undiluted or – if heavy contamination was expected – diluted liquid on Kings Agar B and for some samples also on blood agar. The plates were incubated at 37°C overnight before reading.

One isolate of *P. aeruginosa *from each contaminated production batch of swabs and all isolates identified as *P. aeruginosa *from the system audit were sent for genotyping and compared with the outbreak strain at one or more of three reference laboratories. The strain of *P. aeruginosa *found in the product batch 47.2001 on 8 April 2002 was defined as the outbreak strain. Isolates of *P. aeruginosa *found in the additional investigation of the production site were not genotyped.

Two of the reference laboratories used a pulsed field gel electrophoresis (PFGE) method developed at St. Olav's Hospital and the third laboratory used an augmented fraction length polymorphism (AFLP) The methods are described elsewhere [19]. The AFLP and PFGE protocols were compared and found to be equal in detecting and discriminating the outbreak strain. If an isolate was not typeable by PFGE because of excessive activity of endogenous endonucleases, it was genotyped with AFLP.

## Results

### Investigation of contaminated product

**Figure 3:**
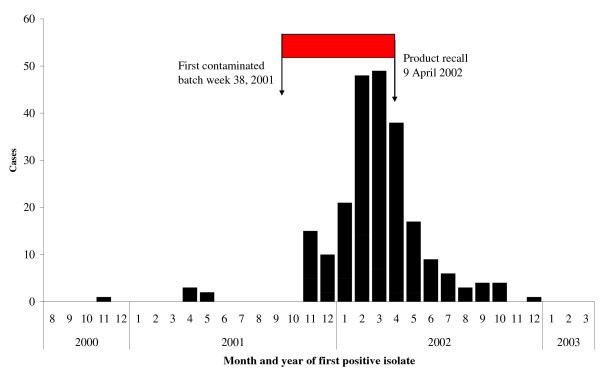
Epidemic curve. Number of cases with the outbreak strain of *Pseudomonas aeruginosa *by month and year of the first positive culture result. Production period for Dent-O-Sept swabs contaminated with *P. aeruginosa*

NIPH received information about stored batches of the Dent-O-Sept swab from 59 of the 65 general hospitals, four other health care services and 20 private persons. Six of the health care institutions reported that they had not purchased the Dent-O-Sept swab and five reported that all remaining batches of the product had been discarded immediately after the cause of the outbreak was made public. The culturing results from 1565 swabs from 149 different batches were reported (Table 1). The outbreak strain of *P. aeruginosa *was detected in 76 swabs from 12 different batches of the Dent-O-Sept swab produced from week 38 in 2001 to week 15 in 2002 when production ceased. These 76 swabs were sent for examination from 13 different hospitals, one private person and from the producer. All genotyped strains of *P. aeruginosa *were identical to the outbreak strain. The outbreak strain was isolated from six patients up to ten months before the production of the first swab found to be contaminated (Figure 3).

**Table 1:** Microbial contamination of different batches of the Dent-O-Sept swab

**Production week and year**	**No of swabs examined**	**No of swabs contaminated with *P. aeruginosa***	**Other microbes isolated and the number of swabs where they were found**
15 2002	12	12	
11 2002	23	1	3: CNS*, 3: *Bacillus *sp., 7: GPMF**,
08 2002	12	0	3: CNS*, 1: Micrococci, 1: Diphtheroid rods, 3: GPMF**
07 2002	14	0	5: CNS*, 3: *Bacillus *sp., 4: Diphtheroid rods, 1: *Staph. aureus*, 1: Micrococci
06 2002	19	2	1: CNS*, 2: *Bacillus *sp., 10: GPMF**, 1: enterococcus, 1: *Staphylococcus *sp.
05 2002	37	2	10: CNS*, 2: *Bacillus *sp., 4: Micrococci, 8: GPMF**, 2: Mixed flora
04 2002	15	10	
51 2001	25	5	7: GPMF**, 1: Colony of mobile rods
50 2001	20	1	5: *Bacillus *sp., 5: Micrococci, 6: GPMF**
48 2001	1	0	
47 2001	32	16	1: CNS*, 9: *Bacillus *sp., 3: Gram-negative rods,
45 2001	11	0	2: *Bacillus *sp., 3: Micrococci
43 2001	16	5	1: Micrococci
42 2001	13	1	1: *Bacillus *sp., 2: Gram-positive cocci, 7: Mixed flora, 1: *Staphylococcus*?
41 2001	28	18	1: CNS*, 3: *Bacillus *sp.
39 2001	15	0	1: CNS*, 2: *Bacillus *sp., 2: Micrococci, 1: GPMF**, 1: *Streptococcus Equisimilis *group G., 1: Mould
38 2001	26	3	4: CNS*, 1: *Bacillus *sp., 1: Micrococci, 1: Yeast
36 2001	21	0	1: Micrococci, 3: GPMF**, 1: Enterocci, 1: Fungus
34 2001	34	0	1: CNS*, 4: *Bacillus *sp., 1: Micrococci, 2: GPMF**
29 2001	2	0	2: CNS*, 2: Micrococci,
27 2001	16	0	1: *Bacillus *sp.
24 2001	21	0	1: *Bacillus *sp., 3: Micrococci, 2: GPMF**
22 2001	11	0	3: CNS*
19 2001	13	0	
18 2001	17	0	
17 2001	18	0	4: CNS*, 1: *Bacillus *sp., 1: GPMF**, 1: α-hemolytic *Streptococcus*, 1: *Acinetobacter *sp.
13 2001	14	0	2: CNS*, 2: *Bacillus *sp.,
12 2001	10	0	1: CNS*, 3: *Bacillus *sp., 1: Gram-negative rods
11 2001	10	0	
09 2001	11	0	2: CNS*, 1: *Bacillus *sp.
07 2001	29	0	5: CNS*, 2: GPMF**
05 2001	21^¶^	0	7: CNS*, 3: *Bacillus *sp., 2: Yeast, 2: *Acinetobacter baumanii*
04 2001	14	0	2: CNS*, 3: *Bacillus *sp.

Total 2000	286	0	26: CNS*, 11: *Bacillus *sp., 3: *Staph. aureus*, 2: Micrococci, 1: Yeast, 1: Mould, 1: *Candida albicans*
Total 1999	164	0	11: CNS*, 7: Bacillus, 2: Gram-positive spore forming, 1: Gram-negative rods, 1: *Staph. aureus*, 1: Yeast, 1: *Pseudomonas *sp. (not *P. aeruginosa*)
Total 1998	181	0	6: CNS*, 7: *Bacillus *sp., 3: Micrococci, 1: GPMF**, 1: Yeast, 2: Mould
Total 1997	139	0	12: CNS*, 2: GPMF**, 7: *Bacillus *sp., 1: Micrococci, 1: Yeast, 1: Mould
Total 1996	63	0	1: *Streptococcus pyogenes*
Total 1995	64	0	3: CNS*, 1: *Bacillus *sp., 1: Micrococci, 2: Enterococci, 11: *Staph. aureus*, 1: *Pseudomonas *sp. (not *P. aeruginosa*)
Total 1994	24	0	
Total 1993	11	0	
1992 and older	52	0	1: CNS*, 5: *Bacillus *sp., 1: Micrococci

* CNS: Coagulase negative staphylococci

** GPMF: Gram-positive mixed flora

^¶ ^For 5 of the examined swabs the wraps had been opened before arrival to the laboratory.

In addition to *P. aeruginosa *another unidentified species of *Pseudomonas *was detected in two swab-batches produced in weeks 6 in 1995 (one swab) and week 22 in 1999 (nine swabs). The strains from the two batches were genotypically identical. In total, more than 250 swabs were found to contain one or more species of micro-organisms, mainly gram-positive bacteria which were predominantly discovered in the earlier batches. Gram-negative rods including *Acinetobacter baumanii *were isolated in swabs produced in 1999 and 2001.

### System audit and additional investigations

The production process of the swabs was described as follows: The foam rubber heads and sticks were glued together manually in private homes of employees who were following moderate hygiene guidelines.

One batch of moisturizing liquid consisted of: Tap water (147 liters), 96% ethanol (3 litres), Glycerol (16 litres) and Vademecum, a commercially available mouth rinse (6 litres). The main ingredients of the mouth rinse are ethanol (44%) and sodium benzoate (5.25%). The final concentration in the Dent-O-Sept moisturizing liquid was calculated to be 2.3% ethanol; 9.3% glycerol and 0.18% sodium benzoate [25].

A new batch of the liquid was prepared in the following way every week of production: The tap water was filled into a large steel tank with a lid on Friday, and then heated to 95°C the following day in an automated but uncontrolled process (Figure 2). On Monday the other ingredients were added and the solution stirred manually with a steel rod.

Packing, moisturizing and sealing of the swabs were done in an automated packing machine, Fuji Wrapper II, FW 3400. The moisturizing liquid was led from an orifice at the bottom of the steel tank through a pipe, via a pump and a dosing valve ending in the packing machine close to the heat sealer. This piping system was approximately 3.5 meters long and a locally made adaptation to the packing machine. The tank and pipe were made of acid resistant stainless steel.

Dry swabs were fed on a conveyer belt and approximately 2.0–2.5 grams of liquid were sprayed into the aluminium-plastic laminate wrap right before heat sealing. Staff tested the bags for air tightness by squeezing ten bags at the time when coming out of the machine before packing.

At the end of each working day, the remaining liquid was kept in the steel tank. Before start of production the next morning the moisturizing liquid was stirred using a steel rod that first was washed with soap and water and then disinfected. On Fridays when the week's production was finished the remaining moisturizing liquid was emptied from the tank. The tank was then flushed with high pressured hot water (> 60°C). Then 30 litres of lukewarm water was added together with 1.5 dL of disinfectant ("FAWA Desinfekt" contains 1–5% by weight of alkyl dimethyl benzyl ammonium chloride, a quaternary ammonium compound). The tank and lid were scrubbed and the solution pumped into the pipes of the packing machine until all moisturizing liquid was assumed to be replaced. After standing for 10–15 minutes the tank was emptied and flushed again with high pressured hot water. Then 30 litres of hot water (73–80°C) was filled in the tank and pumped through the pipes of the packing machine until the disinfectant was assumed to be rinsed out. The producer had no quality assurance routines for checking temperatures, composition of the moisturizing liquid during the week or effect of the cleaning and disinfection process.

A sample taken from the end capillary nozzle in the production facilities was negative on initial culturing, but growth was noted and the outbreak strain of *P. aeruginosa *was identified after it had been incubated in a growth broth. *P. aeruginosa *was not detected from other points along the production line, the drain or at other points sampled in the production room. However, the outbreak strain was detected in packed samples of the product batch 15.02 stored on the premises.

The system audit concluded that the production deviated from the existing regulations in several areas:

• The production process, including the recipe for Dent-O-Sept, did not ensure that the product had the qualities and properties stated by the producer nor that the risk of contamination was avoided or reduced to a minimum.

• Neither the boxes nor wraps of the Dent-O-Sept gave the user the necessary information. The CE (Communauté Européenne) marking was unjust because the producer's declaration of conformity with the regulations, including the risk analysis, was poorly based and documented. The technical documentation did not give a third party a basis for assessing whether the device was in accordance with the demands of the regulations.

• The producer did not comply with the obligation to report defects and deficiencies in medical devices to national health officials and had not adequately followed up errors in the production demonstrated in an external review in 1999.

The additional investigations revealed there was a wide variety in the bacterial load of *P. aeruginosa *in swabs produced on the same day and even at the same time of day ranging from 50 to 10000 CFU per mL liquid in the five positive swabs of the 16 examined (Table 2).

**Table 2:** Quantitative analysis of bacterial load of *P. aeruginosa *of the product taken from different boxes produced at different times on two consecutive days

Box	Sample no	Prod. date	Prod. time	CFU of *P. aeruginosa *per mL
A	1	08.04.2002	-	10000
	2	08.04.2002	-	5000
	3, 4	08.04.2002	-	0
B	1–4	08.04.2002	-	0
C	1–4	08.04.2002	-	0
D	1	08.04.2002	-	50
	2	08.04.2002	-	1000
	3	08.04.2002	-	70
	4	08.04.2002	-	0

E	1	08.04.2002	08.30	630
	2	08.04.2002	08.30	230
	3	08.04.2002	08.30	2900
	4	08.04.2002	08.30	1100
	5	08.04.2002	08.30	100
F	1–5	08.04.2002	14.00	0
G	1–5	09.04.2002	08.30	0

*P. aeruginosa *was cultured from the steel rod after both of the two attempts of cleaning and disinfection were performed. *P. aeruginosa *was also cultured from the blue connecting pipe and the level measuring device. Water samples taken from the rubber hose on two separate dates yielded >300 and 1400 CFU of *P. aeruginosa *per 250 mL of water. On a third date water samples showed 140 and 170 CFU of *P. aeruginosa *per 500 mL of water after 15 minutes of flushing and 55 and 66 CFU of *P. aeruginosa *per 500 mL of water after 45 minutes. *P. aeruginosa *was not detected in water from the tap after removing the rubber hose or from any other water tap on the production site or nearby premises.

### Moisturizing liquid and main disinfectant

No bacteria were detected in any of the ingredients for the moisturizing liquid. When the outbreak strain of *P. aeruginosa *was added to the Dent-O-Sept solution and to the two concentrations of the disinfectant we observed a 6 log reduction in 15 minutes and for the 1:10 diluted Dent-O-Sept solution a 6 log reduction after 3–6 hours (Table 3). For the reference strain (ATCC 9027) there was a 6 log reduction in 15 minutes for all four liquids.

**Table 3:** Antimicrobial effect of liquids on ca. 10^6 ^CFU per mL of *Pseudomonas aeruginosa *added. Number of CFU per mL solution at time intervals

**The outbreak strain of *P. aeruginosa***						
**Liquids**	**Sample taken immediately after adding**	**15 min.**	**3 hours**	**6 hours**	**24 hours**	**Reduction of CFU after exposure**

Control. Peptone water	1,0 × 10 ^6 ^CFU/mL	Not done	Not done	1,0 × 10^6 ^CFU/mL	Not done	No significant change in CFU after 6 hours
Dent-O-Sept solution	No growth in sample diluted 1:100 *	1 CFU/mL	No growth	No growth	No growth	6 log reduction in 15 min.
Dent-O-Sept solution diluted 1:10	1 × 10^6 ^CFU/mL	1–5 × 10^3 ^CFU/mL	< 10 CFU/mL	No growth	No growth	2–3 log reduction in 15 min. 6 log reduction in 3–6 hours

**The reference strain ATCC 9027 of *P. aeruginosa ***						

**Liquids**	**Sample taken immediately after adding**	**15 min.**	**3 hours**	**6 hours**	**24 hours**	**Reduction of CFU after exposure**

Control. Peptone water	1,5 × 10 ^6 ^CFU/mL	Not done	Not done	1,4 × 10^6 ^CFU/mL	Not done	No significant change in CFU after 6 hours
Dent-O-Sept solution	No growth in sample diluted 1:10 *	1 CFU/mL	No growth	No growth	No growth	6 log reduction in 15 min.
Dent-O-Sept solution diluted 1:10	Ca. 10^6 ^CFU/mL	No growth	No growth	No growth	No growth	6 log reduction in 15 min.

* Not tested in undiluted sample

## Discussion

### Contamination of the swabs

We have described how the Dent-o-sept mouth swabs were contaminated with *P. aeruginosa *during production and consequently caused a major outbreak. Genotypically identical strains of *P. aeruginosa *were isolated from 231 patients. The same genotype of *P. aeruginosa *was detected in batches of Dent-O-Sept swabs produced from week 38 in 2001 while the first patient with the outbreak strain was sampled in December 2000, ten months earlier. This can be coincidental or due to the fact that *P. aeruginosa *in almost all instances is an obligate aerobe and will succumb over time as the oxygen inside the wrap is used [26,27]. Swabs from 12 different production weeks were found to be contaminated. Oxygen depletion within the wrap may have lowered the ability to detect *P. aeruginosa *especially in older swabs where it had been present. We did not recommend the laboratories to use a growth broth when culturing the swabs. This may have decreased the culturing sensitivity and prevented us from detecting *P. aeruginosa *in more batches of the swabs.

The finding of genotypically identical strains of another species of *Pseudomonas *four years apart indicate that also other bacteria could remain in the production line for years. The multitude of gram-positive bacteria found in many of the swabs examined and which survived many years in the wrap, more probably stem from the dry part of the production. Although most of the micro-organisms are harmless for healthy people, they can pose a risk to the susceptible patient. This should be kept in mind when using clean but non-sterile products on the most vulnerable patients.

### *Pseudomonas aeruginosa* in the production line

*P. aeruginosa *was detected throughout the wet part of the production of Dent-O-Sept swabs. Due to the direction of flow of the liquids through the production line one can assume that the rubber hose was the first to be contaminated either from the tap water or from direct contact with the orifice of the rubber hose by contaminated hands or objects. The chipped rubber hose gave ample possibilities for permanent contamination of *P. aeruginosa *and other organisms. The large number of swabs contaminated with *P. aeruginosa *and the finding of the outbreak strain in the production line makes it highly unlikely that the swabs were contaminated after sealing the wrap.

*P. aeruginosa *does not grow in the moisturizing liquid used for the Dent-O-Sept swab. In fact even when added to a 1:10 diluted solution, the bacteria were rapidly killed. Still, bacteria were detected in a number of swabs and in the wet part of the production line. Interestingly the quantity of bacteria varied greatly between swabs even when produced on the same date and time of day. The most plausible explanation for these facts is biofilm formation in the production line.

*P. aeruginosa *is well known to form biofilms [28-30]. Biofilms are structured, specialized communities of adherent microorganisms encased in a complex extrapolymeric substance matrix [28] which can form on any surface although some surfaces are known to retard adherence [29]. When a biofilm is formed and reaches a critical mass the quorum sensing molecules excreted alter many of the functions of the bacteria, including slowing its metabolism and increasing the production of a glycocalyx matrix [27,31]. These and other factors reduces the bacteria's susceptibility to antibiotics and disinfectants [29,30]. It has been shown that *P. aeruginosa *can reappear after biofilms on polyvinylchloride pipes have been exposed to a variety of disinfectants for seven days [32]. To eradicate the viable bacteria in a biofilm heat is preferred. Alternatively mechanical removal or the use of oxidative biocides to slowly dissolve the biofilm matrix [30] are suggested. Once a biofilm has formed and matured it can spread to new locations either through single cell dispersal or the shedding of clumps of biofilm [27-29].

Biofilm formation in the wet part of the production and the shedding of clumps of biofilm into the bags with the Dent-O-Sept swab can explain why *P. aeruginosa *could survive in the production line and the uneven distribution of bacteria in swabs. The hot water used to disinfect did probably not reach all areas at required temperatures and the disinfectant might not have had an adequate effect on the biofilm in all areas.

The mass media gave much attention to the dry part of the production taking place in private homes. Although this part most probably did not play a part in the *Pseudomonas *outbreak, it is very likely that it could contribute to the contamination with gram-positive bacteria.

### System audit

Medical devices are strictly regulated in Norway and the legislation is in accordance with EU regulations [20]. The system audit revealed several violations of the regulations. The producer knew there had been problems with the production earlier due to complaints from customers of some discoloured swabs and had ordered an external review in 1999. But he had not complied with all the recommendations given and he could not document that a risk analysis had been performed. The police started an investigation of the producer but decided not to press charges. It is also worth noting that the producer was certified after the ISO 9002 standard (Quality systems – Model for quality assurance in production, installation and servicing) by Det Norske Veritas (DNV). This large outbreak and the investigation of the product and production have revealed the necessity to adhere to the rules and the fatal consequences that can occur if they are not.

### Health care institutions

During the investigation, many health care institutions discovered severe weaknesses in their purchasing and storing systems, including finding batches stowed away that were ten years old and more. Although more important for other medical devices like sterile equipment, lengthy storage can also have influenced the bacterial content of the Dent-O-Sept swabs. The first patient with the outbreak strain of *P. aeruginosa *was sampled ten months before the first swab found to be contaminated was produced. This can be coincidental or due to the fact that *P. aeruginosa *in almost all instances is an obligate aerobe and will succumb over time as the oxygen inside the wrap is used [26,27]. Other bacteria like gram-positive cocci can survive for years under these conditions.

The swab was intended for single use only. However, some health care personnel reported that the swab was sometimes stored in a glass of water on the patient's night stand and reused. This practice may have substantially increased the bacterial load the patients were exposed to as indicated in a report [33]. The extent of this malpractice and the impact it had on the size and seriousness of the outbreak is difficult to assess.

Great responsibility is placed upon the purchaser in the health care system to ensure that the products bought are not harmful for the patients. It is also important to have quality assurance systems that discover flaws in medical devices and that all errors are being reported. Partly as a consequence of this outbreak the Norwegian health authorities have revised their regulations for medical devices and are currently improving the reporting system when serious incidents or harm occur. From 2003, medical practitioners in Norway have been obliged to immediately warn the Norwegian Institute of Public Health of cases of infectious diseases suspected to be caused by contaminated medical devices.

The Dent-O-Sept mouth swab belongs to Medical device Class 1, which includes most non-invasive medical devices according to the European Council Directive 93/42/EEC [20]. The devices must, when used, "not compromise the clinical condition or the safety of patients". "The devices and manufacturing processes must be designed in such a way as to eliminate or reduce as far as possible the risk of infection to the patient, user and third parties." Beyond this, the directive does not specify the microbial quality of the product. In comparison pharmaceutical preparations for use in the respiratory tract are according to the European Pharmacopoeia classified in a Category 2 where the absence of *Pseudomonas aeruginosa *needs to be documented [34]. Medical devices and products have often been linked to pseudomonas outbreaks [3-9]. This outbreak has necessitated a reassessment of the guidelines for preventing infections in critically ill and otherwise susceptible patients. Oropharyngeal colonization is important for the development of ventilator-associated pneumonia (VAP) [35] and oral care may prevent pneumonia [36], but few have addressed whether oral products other than ventilator or nebuliser equipment need to be sterile or high-level disinfected for this patient group [37]. *Pseudomonas aeruginosa *is the most common gram-negative bacteria causing VAP [38]. We believe that sterility is not necessary for such products, but only documented quality-controlled, high-level disinfected items, including tap water and moist products, should be used in the oropharynx of this susceptible patient group. It is, however, also necessary to underline that health care institutions have to use also such seemingly simple devices properly and that they in this connection under no circumstances are reusing devices that are intended for single-use.

## Conclusion

In conclusion, the Dent-O-Sept swabs that in 2001–2002 caused one of the largest ever described outbreaks of *Pseudomonas aeruginosa *infection in Norway were contaminated during production when the swabs were sprayed with a purportedly antiseptic moisturizing liquid. Although the liquid was produced each week, it was contaminated with *Pseudomonas aeruginosa *possibly in the form of shedded biofilm from the improperly disinfected mixing tank or associated pipes or hoses. Probably several tens of thousands swabs from a period of at least one and a half years were contaminated and then used in the Norwegian health care system.

## Competing interests

The author(s) declare that they have no competing interests.

## Authors' contributions

BGI headed the outbreak investigation and the conception, drafting and revision of the manuscript. HME performed the logistics in collecting and collating the results from the microbiological examination of the swabs. GB headed the additional investigation of the production site. KH was in charge of the system audit. TJ participated in the outbreak investigation, adapted the PFGE method to detecting the outbreak strain and analyzed many of the samples. EE was in charge of the analysis and antimicrobial effect of the ingredients for the moisturizing liquid. JL participated in the microbiological aspects of the outbreak investigation and performed some of the analyses. PA was over all in charge of the outbreak investigation and participated in the conception, drafting and revision of the manuscript. All authors read and approved of the final manuscript.
